# Editorial

**Published:** 1901-09-15

**Authors:** 


### EDITORIAL.

One of the most dramatic incidents connected with the recent meetings of the National Associations at Milwaukee, was the appearance of Mr. J. H. Worman, the United States consul at Munich, Germany, with a wheelbarrow load, almost, of documentary evidence, and a most earnest and fiery speech, with which he convinced all present that the American dental degree of Doctor of Dental Surgery was being rapidly and woefully discredited in the German Empire, because of the fraudulent and extensive distribution of diplomas in all parts of Germany by at least two disreputable institutions located in this country. And what was even worse, he presented evidence which indicated that one of our State examining boards had certified to the reputable standing of the schools, thus recognizing these fraudulent graduates, and issued permits to them to practice; and also that the consul for the German Empire, located in one of our large cities, had certified to the standing of the schools in question. Thus it would appear that all diplomas, issued by even the best schools in this country, were looked upon by the German people as having the same value. The two organizations raised a fund of over three thousand dollars and appointed committees to ferret out the frauds and, if necessary, take such steps as may be necessary to ascertain who is responsible, with the idea of punishing the guilty parties and stopping this nefarious traffic. Since the adjournment of the Association we learn that a former member of the Illinois State Board of Examiners has been arrested and is being held for trial for fraudulently issuing licences to practice to graduates of unrecognized schools. The committee is evidently at work, and it is to be hoped that this matter may be quickly adjusted, so that there may no longer rest upon our system of professional education the odium of fraud and incompetency.

---

From an editorial in the August *Cosmos* we learn that the French Association for the Advancement of Science has created a new section designed particularly for the biological study of odontology. The editorial says: "As we understand it, the newly created section on odontology will not primarily concern itself with the study of odontology from the point of view of the dental practitioner." And also that "It is not dentistry as a profession, but dentistry as a science in its broader conception as odontology, which will form the objective basis of study by the section." The editorial asks us to, in effect at least, give three cheers for the great honor conferred. Well, cheers don't cost much, and we are willing to second the motion, and a tiger, too, if necessary. But now that we have done it, we wonder why we have done it. Of course, we recognize the great value of the purely scientific investigation of any and all subjects in the discovery of truth. But what is truth good for that has no practical application? It is very likely that all the facts brought to light by this great scientific organization will be made use of by a practical profession. But we are wondering if this knowledge would be vitiated if developed by scientists who had a vital connection with a practical application of it.

THE volume of transactions of the National Dental Association for the meeting held July 10, 1900, has just been issued. It is about half the size, in pages, of the 1899 volume. The lateness of issue is due entirely to the neglect of the publishers. It is printed and bound in the usual style and well done. The delay in publication has caused the publication committee to make a change of publishers, and it is promised that this year's proceedings will be in the hands of members within three months.

---

THE editor of *Items of Interest*, in commenting on dental educational matters in the August issue, makes the following suggestion as a plan for ranking dental educational institutions:

"Appoint a committee to investigate the standard of instruction maintained in schools known to be the best. Let all schools who maintain such standard be recognized as first class. If other schools must have recognition, list them as reputable, but second class. We have first and second-class academic colleges. Is it reasonable to suppose that all the dental schools stand on a par, as recognition by the Faculties Association at present implies? Classify the colleges, and inevitably those in the second grade will either improve or disband, an advantage in either event."

We guess the editor never attended a meeting of the National Association of Faculties. We should be be pleased to be present when such a proposition was made; or, still better, when the executive committee made its report. Oh, my!

